# Indoor Air Pollution Increases the Risk of Lung Cancer

**DOI:** 10.3390/ijerph19031164

**Published:** 2022-01-21

**Authors:** Ke-Cheng Chen, Shih-Wei Tsai, Ruei-Hao Shie, Chian Zeng, Hsiao-Yu Yang

**Affiliations:** 1 Division of Thoracic Surgery, Department of Surgery, National Taiwan University Hospital, Taipei 100, Taiwan; cskchen@gmail.com; 2 Department of Surgery, National Taiwan University College of Medicine, Taipei 100, Taiwan; 3 Institute of Environmental and Occupational Health Sciences, National Taiwan University College of Public Health, Taipei 10055, Taiwan; shihweitsai@ntu.edu.tw; 4 Department of Public Health, National Taiwan University College of Public Health, Taipei 10055, Taiwan; 5 Green Energy & Environmental Research Laboratories, Industrial Technology Research Institute, Hsinchu 31040, Taiwan; rueihaoshie@itri.org.tw; 6 Institute of Occupational Medicine and Industrial Hygiene, National Taiwan University College of Public Health, Taipei 10055, Taiwan; michellzeng0912@gmail.com; 7 Department of Environmental and Occupational Medicine, National Taiwan University Hospital, Taipei 100, Taiwan

**Keywords:** indoor air pollutants, bioaccumulation, combustion sources, pleural fluid, exposure assessment, machine learning

## Abstract

(1) Background: Cooking and burning incense are important sources of indoor air pollutants. No studies have provided biological evidence of air pollutants in the lungs to support this association. Analysis of pleural fluid may be used to measure the internal exposure dose of air pollutants in the lung. The objective of this study was to provide biological evidence of indoor air pollutants and estimate their risk of lung cancer. (2) Methods: We analyzed 14 common air pollutants in the pleural fluid of 39 cases of lung adenocarcinoma and 40 nonmalignant controls by gas chromatography-mass spectrometry. (3) Results: When we excluded the current smokers and adjusted for age, the adjusted odds ratios (ORs) were 2.22 (95% confidence interval CI = 0.77–6.44) for habitual cooking at home and 3.05 (95% CI = 1.06–8.84) for indoor incense burning. In females, the adjusted ORs were 5.39 (95% CI = 1.11–26.20) for habitual cooking at home and 6.01 (95% CI = 1.14–31.66) for indoor incense burning. In pleural fluid, the most important exposure biomarkers for lung cancer were naphthalene, ethylbenzene, and o-xylene. (4) Conclusions: Habitual cooking and indoor incense burning increased the risk of lung adenocarcinoma.

## 1. Introduction

Indoor air pollution is considered to be an important environmental risk factor for lung cancer in nonsmoking Chinese women. Although the prevalence of cigarette smoking and secondhand smoking among women in Taiwan is low [1], the incidence of lung adenocarcinoma among women in Taiwan is still rising [2]. Incense burning and cooking were suspected to be important indoor air pollutants for lung cancer in Chinese women [3]. Adenocarcinoma is the primary histological type of lung cancer associated with air pollution [4], especially among women who never smoke [5]. Although the International Agency for Research on Cancer (IARC) classified outdoor air pollution and particulate matter (PM) from outdoor air pollution as carcinogenic to humans (IARC Group 1) in 2013, the evidence between indoor air pollution and lung cancer is still insufficient. Currently, no research has provided biological evidence of air pollutants in the lungs to support the association between indoor air pollution and lung cancer.

Exposure assessment of air pollution remains a significant challenge for environmental epidemiology. Polycyclic aromatic hydrocarbons (PAHs), benzene, toluene, ethylbenzene, and xylene (BTEX) are common pollutants of cooking and incent burning [6]. Although many methods have been developed to model air pollution [7], air pollutants are produced by many sources and have substantial time and spatial variations. Since the lung is the main target organ for the health effects of air pollutants, it is suitable for measuring the concentrations of air pollutants in the lung. The pleural space is located between the lung and the thoracic cavity. Inhaled air pollutants dissolve and accumulate in the pleural fluid through pulmonary microcirculation [8]. Pleural fluid enters the pleural cavity through the lung interstitial fluid and pleural capillaries [9]. Analysis of pleural fluid may be used to measure the internal exposure dose of air pollutants in the lungs.

**Figure 1:**
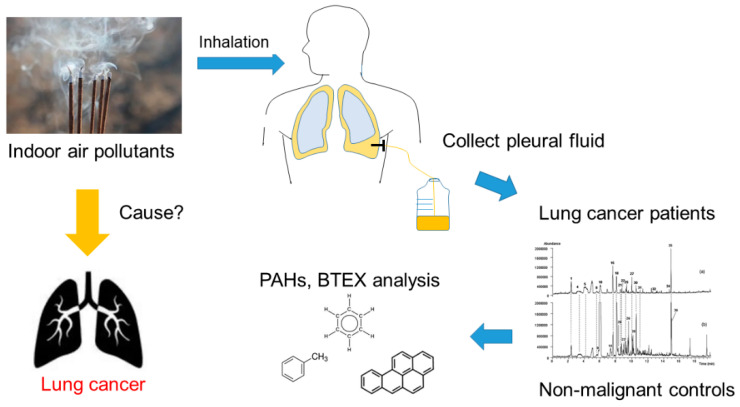
Research theoretical framework. The above figure shows the research hypothesis to find the cause of the rationale between indoor air pollutants and lung adenocarcinoma. PAHs: polycyclic aromatic hydrocarbons; BTEX: benzene, toluene, ethylbenzene, and xylene.

The objective of this study was to assess the association between indoor air pollution and lung cancer (Figure 1). This study used a traditional multivariate regression model to assess the risk of indoor air pollutants and applied machine learning statistical methods to calculate the probability of lung cancer caused by environmental pollutants.

## 2. Materials and Methods

### 2.1. Participants

We conducted a case-control study at the National Taiwan University Hospital from April 2018 to June 2019. We recruited patients with lung cancer and nonmalignant patients with pleural effusion who underwent thoracentesis. Patients with primary adenocarcinoma confirmed by physicians and histological reports were enrolled in this study.

### 2.2. Exclusion Criteria 

Subjects were excluded from the statistical analysis if they had squamous cell carcinoma lung cancer, small-cell lung cancer, metastatic lung cancer from another site, or cancer in other sites. Pregnant women and young people under the age of 20 years old were excluded from the study.

#### 2.2.1. Medical, Occupational and Environmental Histories

We obtained medical histories from patient medical records; histories included tumor stages, medication, imaging findings, serum lactic dehydrogenase (LDH), sugar, total protein, white blood cells (WBCs), blood urea nitrogen, creatinine, alanine aminotransferase, pleural fluid LDH, red blood cells, pleural fluid cytology results, and pathological reports. A face-to-face interview was carried out to obtain detailed occupational and environmental histories. We listed 43 high-risk occupations for lung cancer in Taiwan [10], including glass manufacturing, semiconductor manufacturing, ceramic manufacturing, pottery processing, rock processing, paint manufacturing, magnet manufacturing, stainless steel manufacturing, nickel alloy manufacturing, chromium metal production, disinfectant manufacturing, coke production, vacuum tube manufacturing, cadmium powder production, printing ink production, electroplating, steel foundry worker, nickel smelter welder, refinery worker, rubber production industry worker, coal tar pitch production, battery production, pigment production, plastic production, colorant production, shipbuilder, textile industry worker, asbestos mining and processing worker, talc mining and processing worker, construction maintenance staff, building demolition worker, mason, stone carver, tile inlay workers, plastering worker, shipyard worker, construction worker, car repair and maintenance mechanic, fireman, painter, hairdresser, medical and dental laboratory worker, and Chinese chef. A reviewer asked the subjects whether they worked in any of these types of jobs; if the answer was yes, they were asked the years they started and ended each position, the cumulative number of years they performed the specific type of job, and the tasks they were involved in. The study obtained cigarette smoking and environmental tobacco smoking histories. We also collected residential and workplace addresses to calculate the distance between their residence and the main street with the Google Maps Geographic Information System.

#### 2.2.2. Sample Collection and Preparation

A physician performed thoracentesis and the drainage of pleural fluid. The pleural fluid was collected in a sterile bottle with a gas-tight syringe (SGE Syringes, Trajan, Victoria, Australia) and transferred to a 10 mL vacutainer tube without anticoagulant (BD Vacutainer Plus Plastic Serum Tubes, Becton Dickinson, Franklin Lakes, NJ, USA) to prevent contamination. The tubes were stored in a refrigerator to maintain the samples at 4 °C before centrifugation. The collected samples were sent to the laboratory and centrifuged within three hours. The pleural fluid was centrifuged at 1500× *g* for 10 min by a refrigerated centrifuge designed for heat-sensitive samples to maintain the samples at 4 °C (Centrifuges 5702R, Eppendorf, Hamburg, Germany). The supernatant was transferred into new vacutainers without anticoagulant and then stored at −80 °C until further analysis. To prevent contamination by environmental air, all procedures were performed in a closed system. We placed a stir bar into a 4 mL glass vial sealed with a Teflon/silicone septum and then filled the vial with nitrogen. The pleural fluid samples were initially thawed at 4 °C. Then, we used a gas-tight syringe to inject 2 mL of pleural fluid into the sealed 4 mL glass vial. All procedures were performed in a closed system to prevent contamination by environmental air.

#### 2.2.3. Pleural Fluid Analysis

##### Instruments, Reagents, and Standards

We analyzed the headspace air of the pleural fluid by gas chromatography-mass spectrometry (GC-MS) using the solid-phase microextraction (SPME) technique. GC-MS analysis was performed on a Hewlett-Packard 6890 gas chromatograph equipped with a 5973 mass-selective detector (Agilent Technologies, Santa Clara, CA, USA) as well as a 30 m × 0.25 mm i.d., 0.25 μm thick DB-5MS column (J&W Scientific, Folsom, CA, USA). In our study, we established a GC-MS program using standard solutions, which included eight PAHs (naphthalene, acenaphthylene, acenaphthene, fluorene, anthracene, phenanthrene, fluoranthene, and pyrene) at 10 μg/mL each in acetonitrile, and BTEX (benzene, toluene, ethylbenzene, p-xylene, m-xylene, and o-xylene) at 1 mg/mL each in DMSO. All standard solutions were obtained from Sigma-Aldrich (Milwaukee, WI, USA). Standard solutions were diluted by phosphate-buffered saline (PBS) (Sigma-Aldrich, Milwaukee, WI, USA). A stock solution containing the two classes of compounds (PAHs and BTEX) was prepared using acetone as the solvent for further dilution. Internal standards (ISs) included benzene-d6 (2000 μg/mL in methanol) and naphthalene-d8 (99 atom % D isotopic purity; 1 g in a glass bottle prepared at a concentration of 2000 μg/mL in dichloromethane). All stock solutions were kept in vials and stored at 4 °C. To overcome matrix effects, in which the matrix coextracted with the analytes can invoke a signal response in MS, we added 100 ng/mL of the IS benzene-d6 and naphthalene-d8 during the extraction process [11].

##### Instrument Analysis

The thawed pleural fluid was analyzed by GC-MS within 24 h after collection. This study followed a validated procedure to measure PAHs and BTEX simultaneously [12]. We modified the extraction time, desorption time, and mass range based on our pilot study. We used a 65 μm PDMS/divinylbenzene (PDMS/DVB) SPME fiber (Supelco, Bellafonte, PA, USA). The SPME fiber was inserted into the headspace of a 4 mL vial containing 100 ng/mL of the IS solutions (benzene-d6 and naphthalene) in acetone-d8 and then exposed for 60 min in an 80 °C oil bath. After extraction, the fiber was inserted into the GC injector for analysis. The thermally desorbed trace components were separated by a capillary column with a helium flow rate of 1.3 mL/min using the splitless mode. The chromatographic analytical column temperature was initially set at 35 °C, with a 10 min hold, ramped to 80 °C at a rate of 20 °C/min, and then ramped to 300 °C (10 min) at a rate of 6 °C/min. For the MS measurement, ionization was executed by the electron impact (EI) method at 70 eV while operating in selected ion monitoring (SIM) mode.

##### Optimization of SPME Extraction Conditions

The isolation process was optimized by selecting the appropriate extraction time to obtain the highest extraction efficiency. The optimum conditions were determined by the sum of the peak areas obtained under different extraction times. Hence, the impact of the extraction time was examined in the test sample at 50 °C for various time durations, i.e., 10 min, 15 min, 20 min, 25 min, and 30 min. The extraction time profiles for the total peak area indicated that a sampling time of 25 min adsorption resulted in the highest total peak area. Based on these experimental results, the optimum extraction condition was established as 25 min.

##### Quality Assurance and Quality Control

We conducted blank tests before the analysis of each batch of samples; the blanks included the following:

(1) Instrument blank–A blank instrument test was performed to confirm that there were no contaminants in the system before performing the sample analysis. Before each batch of samples, we ran the GC-MS instrument without any substances to confirm that there were no contaminants in the device.

(2) Reagent blank–To verify that the reagents (PBS) used in the analysis did not contain any compounds in our standard solution, we performed a blank reagent test. We analyzed the reagent (PBS) without any compounds in each batch of samples.

The blank instrument and reagent tests showed that there was no contamination in the system.

##### Method Validation

Targeted compounds were added to PBS at known concentrations to determine the range of quantification. Because pleural effusion fluid does not have an associated artificial sample, we chose a similar biological matrix to serve as the control for the pleural effusion fluid [11]. After the qualitative analysis of our target compounds, the IS was used to calibrate the benzene-d6 IS for the quantification of BTEX, and the naphthalene-d8 IS for the quantification of the PAHs. A fixed amount of IS was added to the sample for detection, and the chromatographic signal with the analyte was divided by the added IS signal. The ratio is extremely relative to the response factor (RF). The integrated area of the sample and the RF were used to determine the concentration. If the corresponding compound had no corresponding IS, then an IS with similar physicochemical properties was used as the basis for correction. The calibration curve was plotted in accordance with the US Environmental Protection Agency (US EPA) standard method. The calibration curve was plotted as the ratio of the peak areas of each volatile organic compound (VOC) to the IS versus the ratio of the concentration of each VOC to the IS. The correlation coefficients (R) of the calibration curves were all above 0.99, and the results showed that the linearity of each compound was acceptable. A value equal to the limit of detection (limit of quantification (LOQ)/√2) was assigned to measurements below the analytical quantification.

### 2.3. Statistical Analysis

First, we conducted a hierarchical cluster analysis to separate clusters and used a heatmap as a visual aid in addition to the dendrogram. The similarity was measured by the Euclidean distance. Age, sex, and cigarette smoking were common confounders for lung cancer. We excluded current smokers and applied multivariate logistic regression to adjust for age, then stratified the data by sex to compare the odds ratios (ORs) in males and females.

We applied four machine learning methods, including decision trees [13], random forests [14], generalized linear models, and neural networks [15] to build the prediction models for lung cancer. A decision tree is best suited for tasks with many features or complexes and nonlinear relationships among features and outcomes [13]. The decision tree utilizes a tree structure to model the relationships among the features and the potential outcomes. It uses entropy to quantify the randomness with a set of class values and finds the splits that reduce entropy. The entropy is specified as follows:

(1) $$\mathrm{Entropy}(S)\;=\sum_{i=1}^{c}-p_{i}\log_{2}(p_{i})$$

For a given segment of data (*S*), term c refers to the number of class levels, and pi refers to the proportion of values falling into class level i. The decision tree uses entropy to determine the optimal feature to split upon, and the algorithm calculates the change in the homogeneity that would result from a split on each possible feature, which is a measure known as information gain. The information gain for a feature F is calculated as the difference between the entropy in the segment before the split (*S*_1_) and the partitions resulting from the split (*S*_2_):

(2) $$\mathrm{InfoGain}\left(F\right)=\;\mathrm{Entropy}\left(S_{1}\right)\;-\;\mathrm{Entropy}\left(S_{2}\right)$$

A random forest is an ensemble consisting of random trees, which are decision trees generated in a specific way to obtain diversity among the trees [16]. The random forest chooses the split for each node to achieve maximum reduction in overall node impurity. The method uses “out of bag (OOB) samples as a validation set to estimate the test error:

(3) $$E{\left(Y-\hat{Y}\right)}^{2}≅OOB_{MSE}=\frac{\sum_{1}^{n}{\left(y_{i}-{\bar{\hat{y}}}_{i,\;OOB}\right)}^{2}}{n}$$

where ${\bar{\hat{y}}}_{i,OOB}$ is the average prediction for the ith observation from the trees for which this observation was OOB. To determine the important environmental variables of lung cancer, we calculated the mean decrease in impurity to obtain the variable importance (VIP) score [17]:

VIP = *OOB_MSE, permutation_* − *OOB_MSE_* (4)

We calculated the area under the receiver operating characteristic curve (AUROC) to assess the prediction accuracy. Statistical calculations were performed using SAS 9.4 software (SAS Institute Inc., Cary, NC, USA), the R statistical language using the rattle package for machine learning [18], the R statistical package for hierarchical clustering, and the online MetaboAnalyst 4.0 (Wishart Research Group, University of Alberta, Canada) (https://www.metaboanalyst.ca, accessed on 13 January 2022) software.3.

## 3. Results

**Figure 2:**
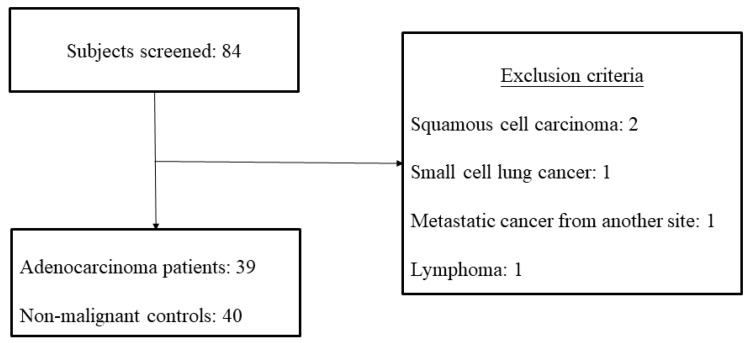
Flow diagram depicting the inclusion and exclusion of the study subjects.

We recruited 43 lung cancer patients and 41 nonmalignant patients with pneumonia, heart failure, pneumothorax, ischemic bowel disease, or Sjogren’s syndrome. After excluding two patients with squamous cell carcinoma, one patient with small-cell lung cancer, one patient with metastatic cancer from another site, and one patient with lymphoma, a total of 79 subjects were included in the final analysis, with 39 cases of lung adenocarcinoma and 40 nonmalignant controls (Figure 2). 

In the case group, the majority of subjects had advanced stage IV cancer. There were no significant differences in smoking status or the distance between the residence and the main street; the prevalence of current smokers was low in both groups. The case group had a significantly higher prevalence of a family history of lung cancer than the control group. Among the environmental factors, the case group had a significantly higher prevalence of habitual cooking at home than the control group (Table 1). The crude ORs of habitual cooking at home and indoor incense burning were 2.73 (95% confidence interval, CI = 0.99–7.50) and 1.73 (95% CI = 0.69–4.36), respectively. When we excluded current smokers and adjusted for age, the adjusted ORs were 2.22 (95% CI = 0.77–6.44) for habitual cooking at home and 3.05 (95% CI = 1.06–8.84) for indoor incense burning. When we further stratified the samples by sex, females had a higher risk than males. In females, the adjusted ORs were 5.39 (95% CI = 1.11–26.20) for habitual cooking at home and 6.01 (95% CI = 1.14–31.66) for indoor incense burning (Table 2).

**Figure 3:**
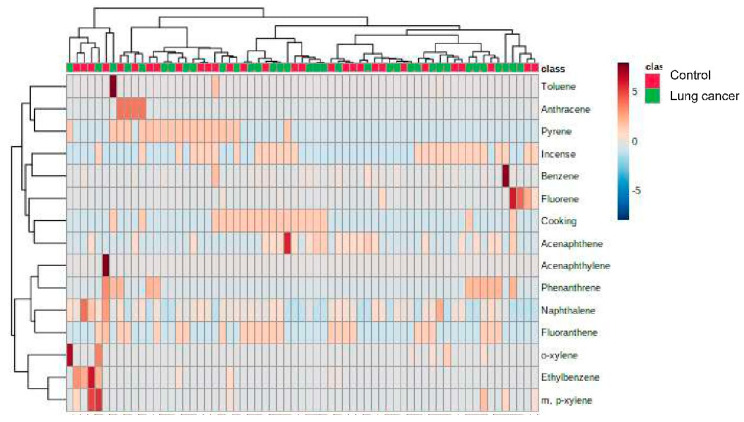
Heatmap of concentrations of exposure biomarkers (PAHs and BTEX in pleural fluid) in lung cancer patients and controls.

**Figure 4:**
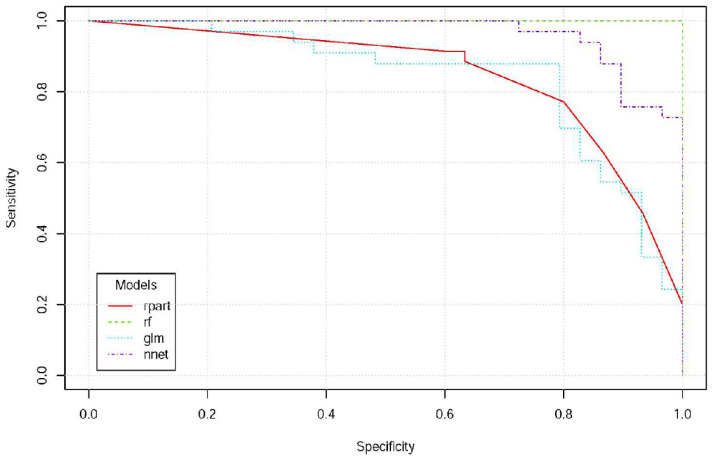
Receiver operating characteristic curves for lung cancer predicted by environmental exposure histories and exposure biomarkers in pleural fluid. We included the prediction variables of environmental exposure histories (lived near the main street, distance to the main street, cigarette smoking, incense burning, essential oil) and exposure biomarkers (PAHs and BTEX in pleural fluid) and used four machine learning algorithms to establish a prediction algorithm. The machine learning algorithms used R packages of the decision tree (rpart), random forests (rf), generalized linear models (glm), and neural networks (nnet). The ROCs ranged from 0.84 to 1.00.

**Figure 5:**
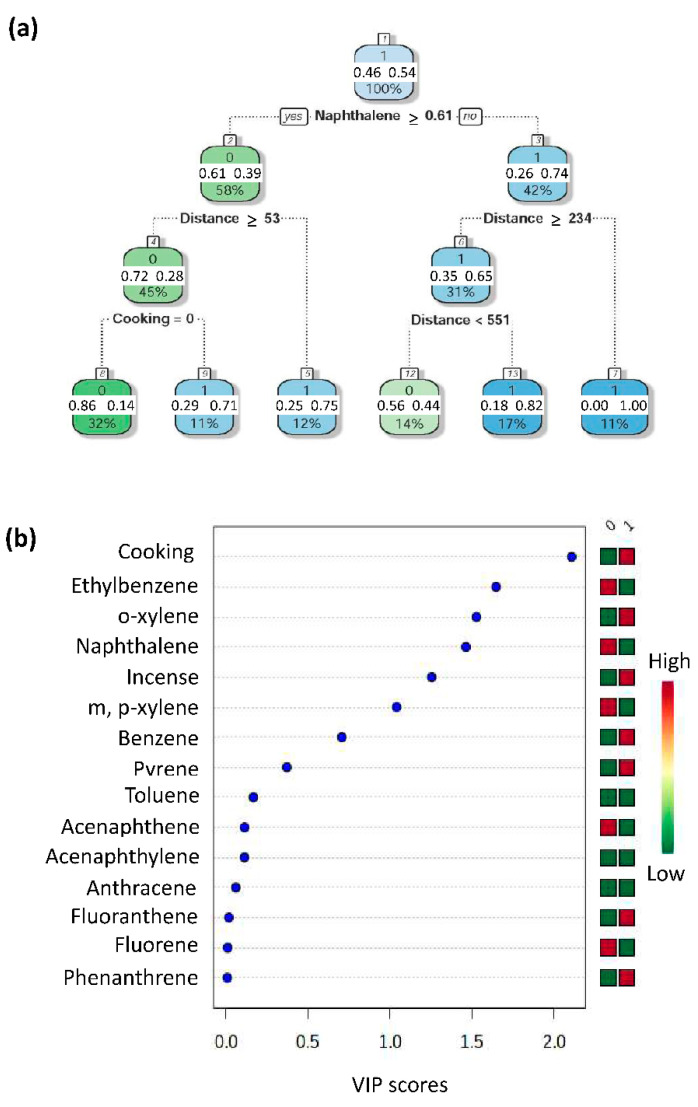
The important environmental factors and exposure biomarkers of lung cancer are determined by (**a**) decision tree and (**b**) random forest models showing the variable importance (VIP) score. By the decision tree, the most important factor in predicting lung cancer is the concentration of naphthalene (≥0.61 ppb) in pleural fluid, followed by the distance between the residence and the main street and habitual cooking at home. By random forest, the most important factor for predicting lung cancer is habitual cooking at home, followed by the concentrations of ethylbenzene, o-xylene, naphthalene in pleural fluid, and then habitual indoor incense burning.

Figure 3 shows the concentrations of exposure biomarkers in lung cancer patients and controls (Figure 3). We included the prediction variables of environmental exposure histories (lived near the main street, distances from the main street, cigarette smoking, incense burning, essential oil) and exposure biomarkers (PAHs and BTEX in pleural fluid) in four machine learning algorithms to establish a prediction algorithm. The mean prediction accuracy of the four machine learning models was 0.91 (standard deviation, SD 0.08) (Figure 4). The decision tree showed that naphthalene in pleural fluid allows us to classify samples into a group of lung cancer. In the random forest model, the most important factor for predicting lung cancer was habitual cooking at home, followed by the concentrations of ethylbenzene, o-xylene, and naphthalene in pleural fluid, and then habitual indoor incense burning (Figure 5).

## 4. Discussion

This study provided evidence that cooking and indoor incense burning increased the risk of lung cancer, especially in females. Environmental exposure history and the measurement of air pollutants in pleural fluid can be used to accurately predict the probability of lung cancer. By combining machine learning technology with analytical science, the public can estimate their health risks of air pollution and take protective measures to prevent lung cancer. Precision preventive medicine is a new concept that guides physicians toward preventive interventions that can work best at the population and individual levels [19]. Precision preventive medicine aims to identify significant environmental risk factors for interventions in the entire community and provide accurate predictive models to identify high-risk individuals in need of primary prevention [20]. By using machine learning techniques, this study took into account individual differences in lifestyle, environmental exposure, and biomarkers that have complex interactions and provided a personalized probabilistic estimate of the lung cancer risk caused by air pollution. This novel method may contribute to the development of precision preventive medicine in environmental health.

This study showed that naphthalene was an important exposure biomarker of lung adenocarcinoma in females. There is no direct evidence in humans that naphthalene causes cancer. However, cancer from naphthalene exposure has been seen in animal studies. Some female mice that breathed naphthalene vapors daily for a lifetime developed lung tumors [21]. We recommend more epidemiological studies to clarify the association between naphthalene and lung adenocarcinoma.

Although blood and urine samples are often used as tools for biological monitoring, they may not reflect the levels of air pollutants in the target organ of the lung because urine and blood contain metabolites of pollutants from all possible routes of exposure. For example, many environmental and occupational studies have measured urinary 1-hydroxypyrene (1-OHP) as a surrogate for PAH exposure [22]. Lai et al. conducted a survey involving Chinese military cooks and found that urinary 1-OHP increased after their work shifts [23]. However, the PAH metabolite levels in urine correlated poorly with personal exposure air sampling [24]. This study directly analyzed the pleural fluid in the lung to assess the internal dose of air pollutants and is thus valuable.

Our study showed that habitual cooking at home is an important risk factor for lung adenocarcinoma in Taiwan. Unlike Western households, which use electric ovens for cooking, Chinese families use woks for stir-frying, steaming, panfrying, deep-frying, poaching, boiling, braising, searing, and stewing. Chinese cooking practices produce high concentrations of oil fumes that contain PAHs, heterocyclic aromatic amines, benzene, and formaldehyde [25]. Different from similar epidemiological studies that provided evidence that the use of coal or brick biomass-fueled stoves increased the risk of lung cancer [26,27], cooking oil fumes are an important source of indoor air pollutants. In Taiwan, a liquid petroleum gas stove is used for daily cooking in most families, and coal is rarely used. Chiang et al. analyzed fumes generated from cooking oils in Taiwan and identified the PAHs dibenz[a,h]anthracene, benz[a]anthracene, and benzo[a]pyrene [22]. Lin et al. compared the incidence of lung cancer between Chinese food chefs and non-Chinese food chefs and concluded that Chinese food chefs, particularly female chefs, had an increased risk of lung cancer [28]. Pan et al. conducted an engineering intervention study to reduce cooking oil fumes in Chinese restaurants in Taiwan. After the installation of a dividing curtain, the oxidative stress biomarkers urinary 8-hydroxy-2’-deoxyguanosine and malondialdehyde decreased in cooks [29]. In Singapore, Hecht et al. conducted a study to quantify carcinogen and toxicant biomarkers in Chinese women who reported regularly doing home cooking compared with women randomly selected from the Singapore Chinese Health Study as controls. The results showed increased exposure to the volatile acrolein, crotonaldehyde, and benzene in Chinese women who regularly cook [30]. We recommend further research into the role of volatile compounds produced during high-temperature cooking of oils as the cause of lung cancer. To prevent hazards from Chinese cooking habits, we suggest that Chinese families improve ventilation while cooking.

Indoor incense burning is a tradition in Chinese culture that shows respect to ancestors. Incense burning is a daily practice. In the morning and afternoon, incense sticks are burned in an incense burner for approximately half an hour. An incense stick is composed of 21% (by weight) herbal and wood powder, 35% fragrance material, 11% adhesive powder, and 33% bamboo stick. Incense smoke (fumes) contains PM, gas byproducts, and many organic compounds. On average, incense burning produces particulates at concentrations higher than 45 mg/g compared to 10 mg/g for cigarettes. The gas products from burning incense include CO, CO_2_, NO_2_, SO_2_, benzene, toluene, xylenes, aldehydes, and PAHs [6]. Kuo et al. measured the total PAHs from incense smoke, and the concentration of PAHs was 147 (±20.6) ng/m^3^ [31]. The current study showed that incense burning increased the risk of lung adenocarcinoma. However, our results are different from the findings among Chinese females in Singapore. Tang et al. conducted a case-control study in Singapore in Chinese females with primary lung cancer and controls. The results showed that the OR of smokers with daily incense or mosquito coil exposure versus nonsmokers without daily exposure was 4.61 (95% CI, 3.41–6.24). However, daily exposure to incense or mosquito coils was not associated with lung cancer among nonsmokers (OR = 0.91; 95% CI, 0.72–1.16), suggesting that smoking played an important role [32]. Another study using the same population-based cohort from Singapore reported that the relative risk of squamous cell carcinomas in the entire respiratory tract among long-term incense users was 1.8 (95% CI, 1.2–2.6; *p* = 0.004) [33]. Even after excluding the effects of cigarette smoking, our study showed that indoor incense burning increased the risk of lung adenocarcinoma. Burning incense produces numerous organic compounds and metals [34,35,36,37]. A recent study found that simultaneous exposure to PAHs and metals had a synergistic effect that increased oxidative damage in coke oven workers [38]. Oxidative stress plays an essential role in the pathogenesis of lung cancer, as it increases the generation of reactive oxygen species (ROS) that cause DNA damage and thus promote lung cancer [39]. We suggest further research to analyze PAHs and heavy metals in Chinese women with lung cancer to clarify the interaction effect.

The occupational hazards of burning incense have been observed in temple workers due to high concentrations and long exposure time. In Thailand, Navasumrit et al. conducted a study on temple workers and found a significant increase in DNA damage; they observed a 2-fold increase in the levels of leukocyte 8-hydroxy-2’-deoxyguanosine (8-OHdG) and DNA strand breaks (*p* < 0.001) [40]. In Taiwan, Chiang et al. assessed human exposure to airborne PM and PAHs during heavy incense burning episodes in temples. The study calculated a 50% probability of exceeding the DNA adduct frequency (DA(f)) ratio for external exposure of Benzo(a)Pyrene and Benzo(a)Pyrene(eq) and a 10% probability of lung tumor due to internal exposure of Benzo(a)Pyrene and Benzo(a)Pyrene(eq). These results implicate that exposure to smoke emitted from mass incense burning may increase lung cancer risk [41]. Since incense burning is a tradition in Chinese culture, we suggest that the public and temple workers should improve ventilation when they burn incense.

Traffic might be an essential source of PAHs in the air [42]. In the Nurses’ Health Study from the United States, the researchers used residential distance to major roadways as a proxy for traffic-related exposure. The study indicated that an increased risk of lung cancer was associated with ambient traffic-related PM exposure, especially among never- and long-term former smokers [43]. Studies in Europe and Canada also used the distance to a main roadway or traffic intensity as a proxy for traffic-related exposure to assess the risk of lung cancer. The results showed that traffic-related air pollution contributed to an increased risk of lung cancer [4,44,45,46,47]. In this study, we compared the distances between home and the main street and did not find a significant difference between the case group and the control group. The study subjects came from Taipei City. We conducted a pilot study to measure the respiratory effect of urban air pollution on cyclists in November and December 2017. We selected a bike lane from a major road and parallel small streets along the major road. The distance of each route was 5 km. Study subjects were randomly allotted to the major road route (*n* = 3) and a small street route (*n* = 3) and rode their bicycles for one hour. With the continuous measurement of the concentration of PM_2.5_ by DustTrak, the mean levels of PM_2.5_ of the high-traffic route and low-traffic route were 7.12 and 5.09 μg/m^3^, respectively. The traffic-related air pollutants in Taipei are not severe compared with other cities. We conservatively estimate that traffic-source air pollution might not be responsible for the increased risk in our case group.

Due to the development of mass spectrometry techniques in environmental analysis, exposure data are often nonlinear, and many variables are highly correlated. The selection of a cluster from the pool of all potential clusters may cause the “Texas sharpshooter phenomenon,” which is a term used to refer to post hoc studies: the Texas sharpshooter shoots first, then draws the target where most bullets have hit [48]. To choose the most influential chemical from multiple chemicals from GC-MS analysis, we applied the machine learning technique to model multiple exposures data that are highly correlated.

In Taiwan, lung cancer is the leading cause of cancer-related death, and the majority of patients (84.1%) are diagnosed in advanced stages (stage III and stage IV) [49]. Therefore, the majority of lung cancer patients enrolled in this study were in an advanced stage, which might have introduced recall bias since lung cancer patients were likely to recall more exposure history. Recall bias might also exist in the family history of lung cancer. The prevalence of first-degree relatives ever having lung cancer was much higher in the case group than in the control group. The ORs of habitual cooking and burning incense at home might be overestimated. The etiology of pneumonia, heart failure, pneumothorax, ischemic bowel disease, and Sjogren’s syndrome are not associated with air pollution. We selected patients with these diseases as the control group to compare the concentration of air pollutants between lung cancer patients and control subjects. The selection of control subjects allows us to explore the exposure biomarkers of air pollutants in lung cancer patients. However, the measured exposure levels of air pollutants may not reflect the causal relationship with lung cancer, especially in cancer epidemiology studies that must consider the long latency until the disease diagnosis. We recommend using a nested case-control study design in established cohorts in future studies. In European countries, indoor radon is a significant risk factor for lung cancer in never smokers [50,51]. We suggest the need for additional epidemiologic research in this area to explore radon and other indoor air pollutants.

## 5. Conclusions

Smoking cigarettes is a significant risk factor for lung cancer. However, smoking is more strongly associated with squamous cell carcinoma than adenocarcinoma. The rising incidence of lung adenocarcinoma in non-smoking females has raised concerns about the role of environmental pollutants in lung adenocarcinoma [52]. We conducted a case-control study to explore the indoor air pollutants of lung adenocarcinoma. This study provides evidence that Chinese cooking practices and incense burning are associated with increased risks of lung adenocarcinoma. For the public, we recommend that Chinese households improve ventilation while cooking and open windows while burning incense.

## Figures and Tables

**Table 1:** Demographic characteristics of the study subjects.

Characteristics	Lung Adenocarcinoma (*n* = 39)	Nonmalignant Controls (*n* = 40)	*p*-Value
Age (years), mean (SD)	66.0 (12.5)	74.5 (15.1)	0.01 *
Male, no. (%)	48 (60.8)	31 (39.2)	0.55
Clinical stage			
I (%)	1 (2.6)		
II (%)	0 (0.0)		
III (%)	4 (10.5)		
IV (%)	33 (86.8)		
EGFR			
Positive (%)	18 (43.4)		
Negative (%)	20 (52.6)		
Individual factors			
Cigarette smoking			
Smoking status			0.56
Nonsmoker, no. (%)	27 (69.2)	31 (79.5)	
Current smoker, no. (%)	1 (2.6)	1 (2.6)	
Former smoker, no. (%)	11 (28.2)	7 (18.0)	
Second-hand smoke (%) ^1^	0 (0.0)	0 (0.0)	
Pack-years, mean (SD)	11.2 (22.5)	7.4 (18.2)	0.42
Occupational factor			
High risk occupation > 10 years (%)	3 (7.7)	1 (2.5)	0.29
Hereditary factor			
Family history of lung cancer (%) ^2^	11 (30.6)	0 (0.0)	0.00 *
Environmental factors			
Lived near main street (<500 m) (%)	36 (92.3)	37 (94.9)	0.64
Distance between home and main street, m (SD)	364.5 (280.9)	335.1 (358.3)	0.71
Lived near factory (%)	2 (5.1)	0 (0.0)	0.15
Habitual cooking at home (%) ^1^	16 (42.1)	8 (21.1)	0.048 *
Habitual indoor incense burning (%) ^1^	18 (47.4)	13 (34.2)	0.24
Habitual use of essential oil (%) ^1^	2 (5.3)	2 (5.3)	1.0

^1^ More than three days a week for more than six months; ^2^ First-degree relative ever had lung cancer; * *p*-value < 0.05.

**Table 2:** Indoor air pollutants and the risk of lung adenocarcinoma.

Risk Factor	Include Current Smoker				Exclude Current Smoker			
	Crude OR	*p*	Adjusted OR ^1^	*p*	Crude OR	*p*	Adjusted OR ^1^	*p*
**All**								
Habitual cooking at home	2.73 (0.99–7.50)	0.05	2.16 (0.75–6.25)	0.15	2.76 (1.00–7.64)	0.05	2.22 (0.77–6.44)	0.14
Habitual incense burning	1.73 (0.69–4.36)	0.24	2.68 (0.95–0.98)	0.06	1.97 (0.77–5.07)	0.16	3.05 (1.06–8.84)	0.04 *
**Female**								
Habitual cooking at home	5.50 (1.15–26.4)	0.03	5.39 (1.11–26.20)	0.04 *	5.50 (1.15–26.41)	0.03 *	5.39 (1.11–26.20)	0.04 *
Habitual incense burning	5.78 (1.12–29.85)	0.04 *	6.01 (1.14–31.66)	0.03 *	5.78 (1.12–29.85)	0.04 *	6.01 (1.14–31.66)	0.03 *
**Male**								
Habitual cooking at home	2.11 (0.46–9.73)	0.34	1.51 (0.24–9.43)	0.66	2.12 (0.46–9.84)	0.34	1.54 (0.25–9.46)	0.64
Habitual incense burning	0.86 (0.27–2.76)	0.80	2.20 (0.48–10.07)	0.31	1.03 (0.31–3.39)	0.97	2.26 (0.56–12.90)	0.22

^1^ Adjusted for age. * *p*-value < 0.05.

## Data Availability

The data that support the findings of this study are available on request from the corresponding author. The data are not publicly available due to privacy and ethical restrictions.
